# The pandemic veneer: COVID-19 research as a mobilisation of collective intelligence by the global research community

**DOI:** 10.1177/26339137221146482

**Published:** 2023-02-13

**Authors:** Daniel W Hook, James R Wilsdon

**Affiliations:** 1 Digital Science, London, UK; 2 Research on Research Institute (RoRI), UCL Department of Science, Technology, Engineering and Public Policy (STEaPP), University College London, London, UK

**Keywords:** COVID-19, meta-research, research funding, research policy, scientific collaboration, collective intelligence, pandemic response, research on research

## Abstract

The global research community responded with speed and at scale to the emergence of COVID-19, with around 4.6% of all research outputs in 2020 related to the pandemic. That share almost doubled through 2021, to reach 8.6% of research outputs. This reflects a dramatic mobilisation of global collective intelligence in the face of a crisis. It also raises fundamental questions about the funding, organisation and operation of research. In this *Perspective* article, we present data that suggests that COVID-19 research reflects the characteristics of the underlying networks from which it emerged, and on which it built. The infrastructures on which COVID-19 research has relied – including highly skilled, flexible research capacity and collaborative networks – predated the pandemic, and are the product of sustained, long-term investment. As such, we argue that COVID-19 research should not be viewed as a distinct field, or one-off response to a specific crisis, but as a ‘pandemic veneer’ layered on top of longstanding interdisciplinary networks, capabilities and structures. These infrastructures of collective intelligence need to be better understood, valued and sustained as crucial elements of future pandemic or crisis response.

## Introduction

In the year following the initial outbreak of COVID-19, the global research community responded with unprecedented speed, and at scale. More than 285,000 scholarly publications were produced in 2020 (around 4.6% of total global research output).^1^ In the same period, more than 9800 grants totalling 6.5 B USD were awarded to support COVID-related research. Several vaccine candidates were developed quickly and moved into clinical trials in the first quarter of 2020 (Li and De Clercq, 2020; Ziady, 2020). But vaccine research was by no means the primary focus: the breadth of research ranged from understanding the mode of propagation of the virus and the effectiveness of protective equipment, to the effects of COVID-19 on vulnerable groups, and the incidence of ‘long COVID’. Secondary topics then rapidly established themselves within the COVID-19 corpus: for example, the effects of lockdown on mental health and wellbeing; impacts on education across different geographies and social groups; immediate and longer-term economic effects.

Two years on, we have seen a further increase in publication volumes, with 8.6% of global research outputs in 2021 related to COVID-19. This is equivalent to all research published on cancer, cardiovascular medicine and non-COVID infectious diseases in the same period combined.^2^ Or for a non-biomedical comparison, equivalent to all research published in 2021 in economics, psychology and studies in human society.^3^

Attempts to analyse the organisation of research knowledge through the lens of collective intelligence were made long before the pandemic (Woolley and Fuchs, 2011). But the response to COVID-19 potentially reflects the largest mobilisation of collective intelligence by the global research community to date, in response to a specific crisis (Mulgan and Chataway, 2020).

Precisely how collective and how intelligent – as opposed to a profusion of disconnected research – remains to be seen. COVID-19-related research is now a multifaceted collection of topics with diverse contributions from almost every discipline. It looks unlikely that this is a short-term publishing bubble that will burst with the passing of the immediate pandemic threat (Brainard, 2020; Callaway, 2020; Hook and Porter, 2020). This raises important questions for the funding, organisation and operation of research, both in response to future crises and societal challenges, and in more conventional modes.

In this *Perspective* article, we explore the extent to which the data indicate that a distinct field, delineated from others, has formed around COVID-19 research. We present data that suggests that COVID-19 research shares many characteristics of the underlying networks from which it has emerged, and on which it has been built. Soft infrastructures for COVID research – as exemplified by highly skilled, flexible research capacity and collaborative networks – predated the crisis, and the speed of response was only possible as a result of sustained investment in research systems, which could be redeployed as required through a bottom-up marshalling of collective intelligence and capability. As such, we argue that COVID-19 research should not be viewed as a distinct field, or as a unique response to a specific crisis, but as a ‘pandemic veneer’ layered on top of longstanding interdisciplinary networks, capabilities and structures.

## Knowledge, networks and nations

Here we present the results of three analyses that show how COVID-19 research is now developing towards an expected or familiar ‘shape’ of research in other areas. We first present a network analysis to highlight the level of influence of each country in COVID-19 research based on inter-country collaborations and subsequent co-authorship of papers. We then show the domestic, bilateral and multilateral splits of coauthorship over the period. Finally, we examine funding acknowledgements in COVID-19-related papers.

**Figure 1:**
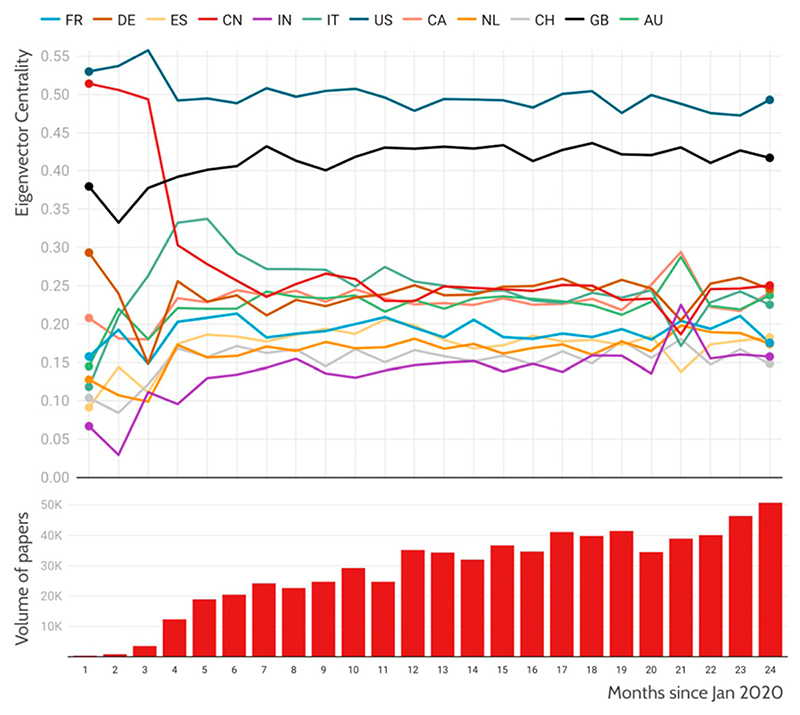
Evolution of influence of each country over COVID publications by month. The upper plot shows eigenvector centrality in the co-authorship graph of COVID publications as a measure of influence. Each country is denoted in a colour shown in the key; eigenvector centrality ranges from 0 to 1; in this plot, the most influential country is the US, with a peak value in month three of 0.55. The lower plot shows volume of COVID publications by month. Data: Digital Science Dimensions.

We begin by calculating the eigenvector centrality of the academic co-authorship graph on a monthly basis, (Figure 1). Month 1 is January 2020, when initial reports surfaced of the COVID-19 outbreak in China. The eigenvector centrality is a network measure that seeks to quantify the importance of different nodes in a network. In the network analysed here, each ‘node’ is a country. Two countries are linked by an ‘edge’ if they have at least one publication that is co-authored by researchers affiliated with an institution in each country.^4^ Each edge can, in addition, be weighed.

In our example here, the weighting is provided by the number of co-authorships. The eigenvector centrality scores the importance of a node (country) in the network by calculating how much it is ‘on the way’ between any two nodes. As we experience in everyday life, friends who are highly connected in our network are highly influential. They gain this influence through their number of connections on one hand and the depth of these connections on the other.

The same is true in the present scenario: countries that are broadly connected to many other countries with high weights on those connections (many co-authored papers in a given year) will be highly influential. Note that it is possible to be influential in many ways and for influence in this sense to produce results that are at first counter intuitive. If a country is a small research producer but is highly collaborative and connected in its relationships, then it may exhibit a large influence over the overall network compared to a large research producer that is highly domestic in its collaborations. The large volume of research from the highly productive but domestically focused country is unread by much of the world as the collaborative links don’t exist to embed that research into the research systems of other countries, whereas a small but highly connected country can influence global research by having its smaller volume of research more widely read through its collaboration or ‘friendship’ network.

The ideal way to influence the network is to produce a lot and also to be highly connected, but for many countries this is not an option. It is also clear that established research have a significant advantage through this type of measure. Similarly, countries that work in English (the *lingua franca* of science), are at a further structural advantage.

In this paper, we study the co-authorship network of COVID-19-related publications as defined by the Dimensions database^5^ (Hook et al., 2020). Each paper is assigned, via the co-author affiliation, to their countries. For example, for a paper with two co-authors, one in China and one in the US, each country will be credited with 50% of the paper and will be linked with a weight of 1. All papers in the dataset are subjected to a similar treatment and the eigenvector centrality is calculated for each monthly time slice. As the volume of papers increases, the stability of the eigenvector centrality increases from month to month. The range of the eigenvector centrality is from 0 to 1 with 1 signifying complete control of the network (not only collaboration on every paper but also being the only actor) and 0 indicating no influence. The United States is the most influential actor in this network as it participates in the largest volume of papers with the greatest diversity of other countries.

We see that in the early months of COVID-19 research, China is highly influential globally, having seen the largest number of initial COVID-19 cases, and hence starting research efforts sooner than others. As the months pass, we see the usual global hierarchy of research influence reasserting itself, with the United States and United Kingdom the most influential collaborators, and China, Germany and other developed research economies quickly falling into an order that closely correlates with the corresponding picture for research in clinical medicine.

**Figure 2:**
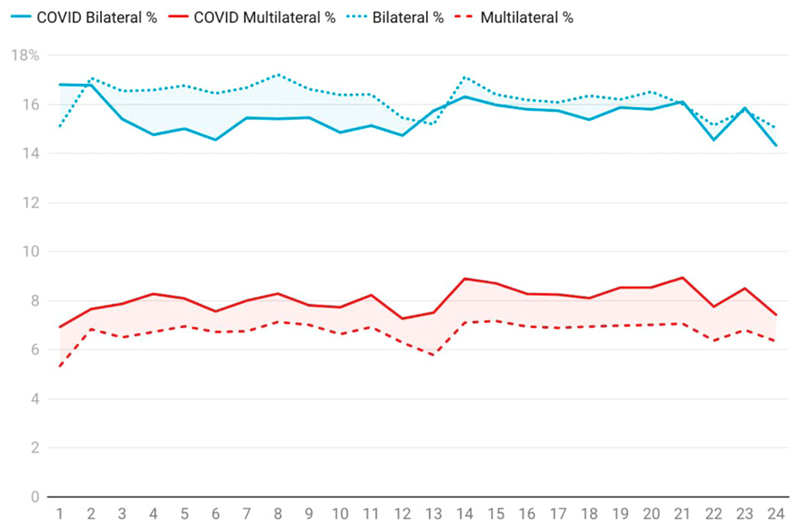
Month-by-mouth development of COVID versus global % of bilateral and multilateral collaboration on publications at a country level. The vertical axis is percentage of output, the horizontal axis is months since Jan 2020. In both cases, the dotted or dashed lines denote the collaboration trend for all papers in all subjects including COVID papers; the solid lines denote the collaboration trends just for COVID papers alone. The number of COVID publications is small in months 1–3 (see Figure 1) and hence the trends are more volatile and less reliable. Data: Digital Science Dimensions.

Turning to collaboration, Figure 2 shows the development of bilateral collaboration (publications involving co-authors from just two countries) and multilateral collaboration (publications involving co-authors from more than two countries) (The domestic, or single country output level, is implicit as the domestic, bilateral and multilateral percentages must sum to 100%). As COVID-19 developed, countries closed their borders, but Figure 2 shows that a few months into the trend (when the number of publications is large enough to be significant) while bilateral collaboration suffered, multilateral collaboration rose. Over the past 2 years, bilateral relationships have mostly recovered to the background level of all research, while multilateral relationships have increased relative to the expected level for all research. It might be argued that all research is not a good proxy for medical research, which is the core of COVID-19 research. However, a comparison of the global levels of bilateral and multilateral co-authorship with that of clinical medicine is in good agreement with the dotted and dashed lines in this plot.

**Figure 3:**
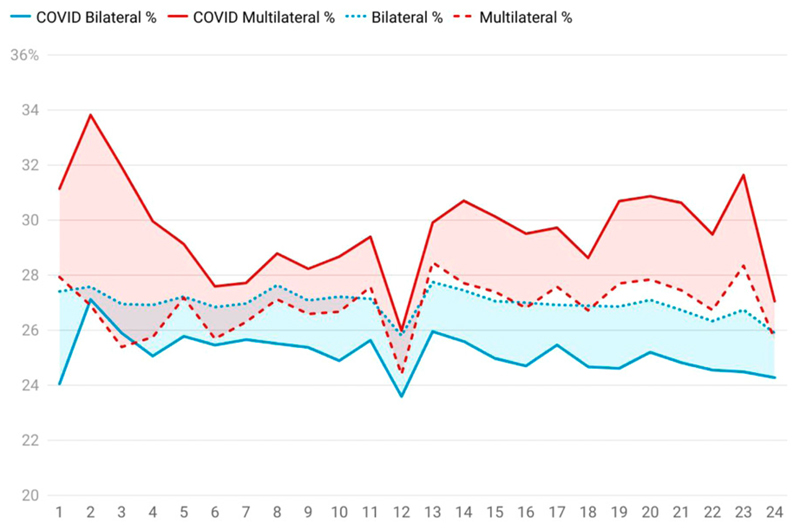
Month-by-mouth development of COVID versus global % of bilateral and multilateral collaboration on publications at an institutional level. The vertical axis is percentage of output, the horizontal axis is months since Jan 2020. In both cases, the dotted or dashed lines denote the collaboration trend for all papers in all subject areas including COVID papers; the solid lines denote the collaboration trends just for COVID papers alone. The number of COVID publications is small in months 1-3 (see Figure 1) and hence the trends are more volatile and less reliable. We note that there appears to be a seasonal effect in December each year. Data: Digital Science Dimensions.

The country-level analysis shown above in Figure 2 can be replicated at an institutional level (replacing countries with institutions irrespective of country in the global analysis performed for Figure 2). In the analysis shown in Figure 3, bilateral collaboration corresponds to coauthorship on a publication where there are precisely two institutional affiliations for co-authors (an edge case here is a single author paper where the author is affiliated with two institutions) and multilateral collaboration corresponds to co-authors on a publication where more than two institutional affiliations are records for co-authors (Again, ‘domestic’ or single-institution papers are implied by the other two curves since single-institution, bilateral and multilateral figures must sum to 100%). Figure 3 gives a sense of the inter-institutional collaboration landscape.

We see that inter-institutional collaboration in COVID-19 research (shown in the solid lines) had been more heavily affected at the beginning of the crisis and, while its movement appears well-correlated with non-COVID-19 research, multilateral collaboration between institutions remains at a higher level than inter-country research (Figure 2), or the global inter-institutional norm (dotted/dashed lines). Bilateral research remains at a proportionally lower level. Hence, it seems that bilateral collaborations have become more multilateral, while single-institution research remains at around the pre-COVID level. We suggest that this continued lack of alignment may be due to specific characteristics of the ‘pandemic veneer’ such as opportunities to bring pre-existing distinct collaborators together on a new problem.

## The online invisible college

Several authors have pointed to the challenges of holding international meetings and maintaining opportunities to develop new collaborations (Dua et al., 2021; Gao et al., 2021; Myers et al., 2020; Schiermeier et al., 2020). It was several months before conference organisers were able to replace larger-scale physical meetings with online alternatives. Now, beyond the standard conferencing platforms such as Zoom and Microsoft Teams, a range of different academic technologies have emerged in this area. These include Morressier’s pivot from focussing on conference posters to online conference management (Morressier, 2020); the emergence of Underline.io as a new platform to host online conferences (Lazinica, 2020); and the launch of Cassyni to scale up seminars from a local to a global audience (Vincent, 2021).

As a result of these and related innovations, a significant portion of what Wagner (2008) described as the ‘new invisible college’ of global research has moved to fully online modes of collaboration. Other activities have been harder or slower to migrate to an online environment. For example, a reduction in the number of serendipitous meetings (such as those that happen over lunch or in the coffee queue at a physical conference) may not show up explicitly in the data, or may be hidden as online meetings and other technological solutions achieve substitutional effects. Figures 2 and 3 do not yet reflect the results of a drop in international travel which, if there is an effect, would take several years to manifest in the data. It is difficult to obtain a precise proxy for conference attendance, as there is no global database of academic conference attendance.

**Figure 4:**
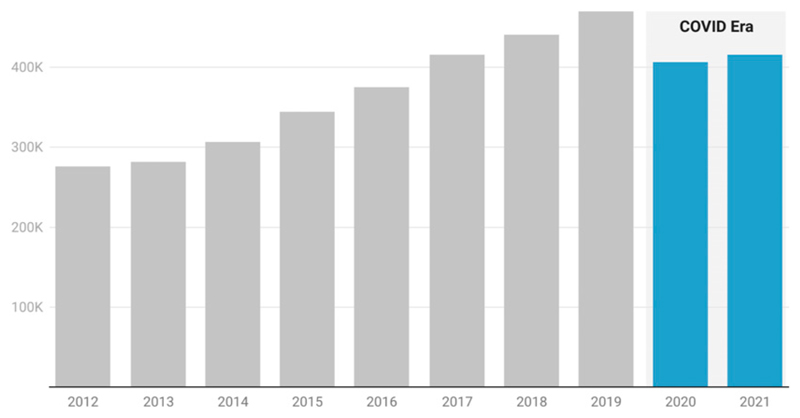
Conference Proceedings publications between 2012 and 2021. During the COVID era, conference proceedings have dropped to around 86% of their pre-COVID levels. Data: Digital Science Dimensions.

Though a lagging indicator, the number of conference proceedings published in 2020 and 2021 gives one signal regarding the effects of COVID-19 on conference participation over the past 2 years. Figure 4 shows the steady increase of conference proceedings articles indexed in the Dimensions database from 2012 onwards. From 2019 to 2020, there is a 16% drop in the number of conference proceedings published, and by the end of 2021, we see a second year of reduction. 16% is arguably a modest drop, given the huge upheaval in conference attendance through the pandemic. In more speculative mode, if we see continued growth in online conferences, recordings of talks may become a new currency of academic value, replacing or adding to formal paper-based proceedings in conference-focused fields (Diner, 2019).

Our final analysis concerns the funding of COVID-19 research and offers the strongest evidence that we are seeing a veneer layered over existing research structures. Since January 2020, more than 850,000 research outputs have been published that refer to COVID-19. Of these papers, only 8.75% acknowledge a specific grant funding source.

Of the 8.75% of papers that do acknowledge a grant, 93% (just over 68,000) refer to grants that either pre-date the COVID-19 crisis or that do not mention COVID-19 in their title or abstract. Only 7% of grant-acknowledging papers mention a grant that is specifically identified as COVID-19-related (just over 5000 papers). Of this 7% of grant-acknowledging papers, 4.5% (around 3215 papers in total) refer to both COVID-19 and non-COVID-19 grants, and 2.5% – around 1785 papers – mention purely COVID-19-related funding.

This picture will change as more targeted COVID-related grants start to become more visible in the acknowledgments of published outputs. But given that new research fields often require some form of funding as a catalyst for consolidation, the low incidence of grant funding acknowledged here (just over a half of one percent of all COVID-related publications since January 2020) suggests that COVID-19 research has been heavily reliant on existing networks, that benefit from core, flexible or block funding, or are supported by background research infrastructures.

## Collective intelligence applied

Viewed together, Figures 1–3 present a picture of research emerging and expanding rapidly through 2020 before reverting to more conventional patterns of influence by leading scientific nations, and levels of collaboration. While policymakers and funding agencies did respond quickly to new COVID-19 research funding needs (Wintour, 2020), the overwhelming majority of publications to date have relied on existing grants or collaborative relationships. It is therefore unsurprising that network statistics such as eigenvector centrality, and more direct analyses of collaborative relationships replicate the pre-pandemic picture.

COVID-19 research has grown at a rate without historical precedent – from 0% of global research output to 8% in under 18 months. There are many reasons for this: the urgency and immediacy of the threat to the health of individuals worldwide; the rapid societal and economic effects that had to be understood and addressed; the strength and availability of existing research networks; and the relatively narrow R&D focus of the initial crisis (though this has since spread across many other challenges).

This is collective intelligence in action. COVID-19 research was like a school of fish moving in synchronicity: a ground-up process that mobilised the skills, capabilities, data, resources and intrinsic motivations of the research community, then combined and targeted these at a specific yet multifaceted problem.

How unusual is this response, and how readily could it be repeated in other areas? One point of potential comparison is the United Nations’ Sustainable Development Goals (SDGs), and their precursor, the Millennium Development Goals (MDGs) (United Nations, 2019). These reflect a larger-scale and more intractable set of societal and research challenges, but the research system response has differed in crucial ways to its response to COVID-19.

Broadly speaking, investments in MDG or SDG-related research have been made gradually, using top-down incentives to encourage researchers to focus on aspects of overlapping, interdependent problems (Wastl et al., 2020). In the 20 years after the MDGs and then SDGs were agreed, research on those topics grew slowly yet steadily to 11% of all global research outputs (Wastl et al., 2020). This in part reflects their breadth: each MDG or SDG engages with a multifaceted set of problems, and often frames a highly interdisciplinary field. So the research response has largely involved building capacity that did not exist before. By contrast, COVID-19 research has relied on leveraging, linking and redeploying existing capacity (Fry et al., 2020; Hook et al., 2020; Wagner et al., 2021).

Despite excitable predictions in early 2020 that the pandemic would reboot research cultures (Apuzzo and Kirkpatrick, 2020) or that the rapid development of COVID-19 vaccines ‘changed science forever’ (Sample, 2020), there is so far limited evidence that fundamental features of research systems have changed significantly as a result of the pandemic (Waltman et al., 2021). Instead, research responses over the past 2 years have leveraged existing relationships and structures in a highly focused way.

We can best describe the development of COVID-19- related research as a global mobilisation of collective intelligence towards a series of related problems, within specified constraints. These constraints include speed (both to benefit the wider local and global community by sharing research, and to benefit from being a research pioneer); a need for openness (with more impetus on open modes of scholarly communication than in earlier crises); reduced mobility (with less opportunity to travel and meet new collaborators); and domestic pressures such as childcare (unevenly distributed, particularly by gender) (Donald, 2020; Matthews, 2020). These factors have all contributed to constructing the pandemic veneer that is COVIDrelated research.

Has COVID-related research produced during the pandemic been of a higher quality or impact than that in other fields, at other times? This is difficult to measure objectively. In terms of impacts, we can perhaps look at uses of COVID-related research in policy commentary as one route to generating some insight. Specifically, we can examine particular tranches of research and resulting levels of policy attention. For example, over the past 2 years, a total of 589 COVID-19-related papers authored or co-authored by a UK-based researcher acknowledged European Commission funding. 536 (or 91%) of these papers garnered policy attention from 320 different policy documents and 20 patents.^6^ (In the same period, UK-based researchers were affiliated with a total of 13,943 papers acknowledging the European Commission – though not associated with COVID-19 – of which 196 received policy attention and 34 patents. So the relative level of attention in policy circles was significantly above normal levels.

## Conclusion: All about infrastructures

As debates intensify over lessons, mistakes and pathways beyond the pandemic, an important discussion is underway about how to ensure greater investment and preparedness in research systems (Lurie et al., 2021), and how to coordinate efforts across different systems (OECD, 2020). Despite some attempts to present new or rapid interventions as decisive (Collison et al., 2021), our analysis suggests that a crucial lesson of COVID-19 is the importance of maintaining flexible research capabilities and infrastructures, which can be directed or redeployed as required.

In research funding, this relates directly to questions of diversity in funding modes, and the need for plural routes – including, in a system like the UK’s, the quality-related block grant, distributed on the basis of the Research Excellence Framework (Costigan and Wilsdon, 2021). It also speaks to longstanding debates about the balance and relationship between innovation and maintenance (Russell and Vinsel, 2018). Describing the infrastructures needed to support collective intelligence, Mulgan notes the need for institutions able to ‘concentrate time and resources over long periods’ (Mulgan, 2018. p.54). Our analysis underlines this point, and suggests that in research policy and funding, these and other soft infrastructures of collective intelligence need to be better understood, valued and sustained as vital elements of future pandemic or crisis response.

## Data Availability

The data for this paper are available on Figshare at: https://doi.org/10.6084/m9.figshare.19454039

## Notes

1 Data from *Dimensions* by Digital Science.

2 Using the National Institutes of Health (NIH) Research, Condition, and Disease Categorization (RCDC) in Dimensions.

3 Using the Australian and New Zealand Standard Research Classification (ANZSRC) in Dimensions.

4 Note that a single researcher can have two or more affiliations and these affiliations can be in different countries. So, two countries can be linked by a single researcher.

5 At http://covid-19.dimensions.ai (accessed 5 May 2022).

6 Data courtesy of altmetric.com
